# Cecal microbiome profile of Hawaiian feral chickens and pasture-raised broiler (commercial) chickens determined using 16S rRNA amplicon sequencing

**DOI:** 10.1016/j.psj.2021.101181

**Published:** 2021-04-20

**Authors:** Sudhir Yadav, Kayla D. Caliboso, Jannel E. Nanquil, Jiachao Zhang, Helmut Kae, Kabi Neupane, Birendra Mishra, Rajesh Jha

**Affiliations:** ⁎ Department of Human Nutrition, Food and Animal Sciences, College of Tropical Agriculture and Human Resources, University of Hawaii at Manoa, 1955 East-West Rd, Honolulu, HI 96822, USA; † Math and Sciences Division, Leeward Community College, Pearl City, HI 96782, USA; ‡ College of Food Science and Technology, Hainan University, Haikou, Hainan province, 570228, China

**Keywords:** broiler chickens, cecal microbiome, feral chicken, Next-Generation Sequencing, pasture-raised chicken, qPCR

## Abstract

This study investigated the taxonomic profile and abundance distribution of the bacterial community in the ceca of feral and pasture-raised broiler (commercial) chickens. Cecal content from feral and commercial chickens (n = 7 each) was collected, and total DNA was isolated. Next-Generation Sequencing (Illumina MiSeq) was performed to characterize the cecal microbiota. Specific bacteria explored were: *Bacteroides, Bifidobacterium, Lactobacillus, Enterococcus, Escherichia*, and *Clostridium*. At the phylum level, 92% of the bacteria belonged to Firmicutes, Bacteroidetes, and Proteobacteria for both feral and commercial chickens. The proportional abundance of Firmicutes was 55.3% and 63.3%, Bacteroidetes was 32.5% and 24.4%, and Proteobacteria was 7.0% and 5.9% in the feral and commercial chickens, respectively. The alpha-diversity Shannon index (*P* = 0.017) and Simpson index (*P* = 0.038) were significantly higher for commercial than for feral chickens. Predictive functional profiling by PICRUSt showed enriched microbial metabolic pathways for L-proline biosynthesis in the feral group (*P* < 0.01). There were a greater percentage of specific bacteria in the feral than commercial chickens, albeit with lower diversity but a more functional microbiota. In conclusion, feral birds have distinguished microbial communities, and further microbiome analysis is mandated to know the specific functional role of individual microbiota. The difference in microbiota level between feral and commercial birds could be accounted to the scavenging nature, diverse feed ingredients, and distinct rearing localities.

## INTRODUCTION

The microbial communities of the gastrointestinal tract, also known as the gut microbiota, are composed of many microorganisms, primarily anaerobic bacteria (Stanley et al., 2013; Yadav and Jha, 2019). The composition of these microbial communities is dynamic and is typically dependent on the host's genetics, environment, and age (Yadav and Jha, 2019). The gut microbiota greatly determines the fate of nutrition, and vice-versa, as nutrition can affect the microbiota, leading to its impact on the overall health of its host (Yu and Morrison, 2004; Jha et al., 2019a). This includes playing a major role in feed digestion, nutrient absorption, and strengthening the immune system (Jha and Berrocoso, 2015). The dynamic gut microbiota and its interactive nature to immune-inflammatory pathways in the gut influence both health status and disease susceptibility (Rodrigues et al., 2020). Since microbial populations are instrumental to their hosts' health, it will help determine what bacteria are dominant and what potential roles they play, either beneficial or non-beneficial. Knowing the healthy gut microbiota composition also provides opportunities to develop strategies to modify it for improving host performance, immunity and improving food safety of meat animals (Yadav and Jha, 2019). Many studies have been conducted with the analysis of the 16S rRNA gene to understand the microbiota of broiler chickens (Stanley et al., 2013). Also, the use of high-throughput next-generation sequencing (**NGS**) helps to understand the gut microbiome better, and knowledge of its interaction with the host body will help design strategies that can potentially improve the health of chickens (Kumar et al., 2018). These recent molecular methods make it possible to obtain a complete census of the microbial community and provide new ways to look at the biological and ecological roles of the microbiota (Shang et al., 2018). These methods have shown interesting results, such as age as an influencing factor in cecal microbiota composition and dysbiosis in feral chickens due to typhlitis in the lumen of chicken gut (Ocejo et al., 2019).

There is very limited or no knowledge of the gut microbiota of Hawaiian feral chickens. In Hawaii, the population of feral chicken is relatively high. The Red Junglefowl was likely to be the first breed brought by the Polynesian settlers (Gering et al., 2015). More recently, European-derived breeds have been brought to Hawaii for food production and cockfighting (Gering et al., 2015). Ultimately, damage from large storms such as Hurricane Iwa and Hurricane Iniki caused the release of these domesticated chickens into the wild overtime. These have survived as self-breeding populations found on all the Hawaiian Islands (Gering et al., 2015). In addition to their variable genetic makeup, feral birds are subjected to much more variation in their environment, which can have a greater effect on their gut microbial profile. Exposure to different microbes occurs through environmental factors such as diet, water, soil, social interactions, and nesting environments (Grond et al., 2018). Waite and Taylor (2015) stated that diet is more strongly influential to the makeup of the gut microbiota than that of host genetics. Moreover, the diet available in the early stage of life has a profound effect on the gut microbiota and overall health of chickens (Berrocoso et al., 2017; Jha et al., 2019b; Zhang et al., 2020). The objective of this study was to define cecal microbial community profiles for both feral and commercial chickens. Studying microbial community profiles for feral and commercial chickens will be instrumental in understanding the breed differences in development, health, digestion, nutrient absorption, and immunity.

## MATERIALS AND METHODS

### Chicken and Sample Collection

All animal handling procedures were done following the approved protocol from the Institutional Animal Care and Use Committee of the University of Hawaii (UH).

Ten four-weeks-old pasture-raised Cornish Rock broiler chickens were sourced from a local farm in Hawaii, where they were raised in a rotational pasture system. The chickens were transferred to the Small Animal Facility (SAF) of UH, where they were kept for a week to acclimatize to the corn and soybean meal-based commercial broiler diet and kept in floor pens with new litter. The chickens were fed ad libitum commercial feed and water to adjust the pastured chickens to a commercial diet. The chickens were monitored regularly to ensure they were in good health for 1 wk. Nine feral chickens (approximately 15–20 wk old) were collected from a public location in Honolulu and were transferred to the SAF, in a separate room, just before sampling. The feral chickens were euthanized by CO_2_ inhalation immediately without access to any feed or water at SAF. Seven feral and seven commercial chickens were randomly selected for cecum sample collection. The chickens were dissected to remove the ceca, which was wrapped in sterile aluminum foil and placed in a whirl pack. The cecum samples were snap-frozen in liquid nitrogen until transferred to a −80°C freezer until further analysis.

### DNA Extraction

DNA was extracted from the mixed cecal content samples using a Repeated Bead Beating Plus Column Method (RBB+C) with the QIAamp DNA Stool Mini Kit (Qiagen Inc., Valencia, CA) as described by Yu and Morrison (2004). Briefly, the cecal contents were thawed gradually on ice and uniformly mixed to create a representative sample of the bacteria found within the entire cecum. Cells were subjected to mechanical beating using silica beads as per Yu and Morrison (2004). Purified genomic DNA was isolated by removing the RNA and proteins using QIAamp Mini spin columns. Extracted DNA was quantified using a GE NanoVue spectrophotometer (Biochrom, Holliston, MA), and quality was determined using 0.8% (w/v) agarose gel electrophoresis.

### 16s rRNA V3-V4 Amplification and NSG

To analyze the metagenomic profile of the feral and commercial chicken samples, the genomic DNA was used to prepare a 16S sequencing library for amplicon high-throughput sequencing according to the standard Illumina protocol (16S Metagenomic Sequencing Library Preparation) (Amplicon, 2013). The V3-V4 hypervariable region of the 16S rRNA gene was amplified using the 16S Amplicon PCR forward primer 5’TCGTCGGCAGCGTCAGATGTGTATAAGAGACAGCCTACGGGNGGCWGCAG; and reverse primer 5’GTCTCGTGGGCTCGGAGATGTGTATAAGAGACAGGACTACHVGGGTATCTAATCC) following Kumar et al. (2018). These primers were used and verified by the manufacturer as the most promising bacterial primer pair for NGS-based diversity studies (Illumina Inc., Hayward, CA). In this primer pair, Illumina adapter overhang nucleotide sequences were also added to the gene-specific sequences following the manufacturer's instructions. The first stage of Amplicon PCR had an initial denaturation stage of 95°C for 3 min, followed by 25 cycles of PCR with denaturation at 95°C for 30 s, annealing at 55°C for 30 s, and extension at 72°C for 30 s. Then a final extension step was performed at 72°C for 5 min.

After the first round of PCR amplification, the amplicons were purified using the Omega Mag-Bind Total Pure NGS (Omega Bio-Tek Inc., Norcross, GA). Purified samples were quantified using the GE NanoVue spectrophotometer and then submitted to the Advanced Studies in Genomics, Proteomics, and Bioinformatics Core at UH for the second phase of Index PCR, PCR clean-up, library quantification, normalization, and sequenced for Illumina MiSeq 300-bp paired-end sequencing.

### Quantitative Real-Time PCR

Quantitative PCR (**qPCR**) is a well-known technique in microbial community analysis, where quantification of the number of target genes is considered. Although relative quantification using the CT method is common in gene expression analysis, it has also been used in microbial studies (Walsh et al., 2011). In this study, qPCR was used to target and determine the abundance of specific bacteria within the feral and commercial samples. Specific primer pairs were used to target genes that are known to the bacteria of interest (Table 1). Target bacteria included *E. coli, Salmonella enterica, Listeria, Lactobacillus acidophilus,* and *Bifidobacterium bifidum*. The target genes included *uid*A (beta-glucuronidase) (Maheux et al., 2009), *tuf* (elongation factor tu) (Maheux et al., 2009), *inv*A (invasion gene) (Galan et al., 1992; Csordas et al., 2004), *iap* (invasion associated protein) (Barbau-Piednoir et al., 2013), and the 16S-23S intergenic region (Matsuki et al., 2003; Haarman and Knol, 2006). Also, a universal bacterial primer set optimized for qPCR, p1 and p2 (Muyzer et al., 1993), was used as a reference gene to normalize the abundance of the bacteria of interest.

**Table 1:** Target organisms and primer pairs used for studies by qPCR.

Organism	Target gene	Amplicon size	Primer
*E. coli*	*uidA* gene	147	**UAL754:** 5′-AAAACGGCAAGAAAAAGCAG-'3 **UAR900:** 5′-ACGCGTGGTTACAGTCTTGCG-'3
*E. coli*	*tuf* gene	258	**TEco553:** 5′-TGGGAGCGAAAATCCTG-'3 **TEco754:** 5′-CAGTACAGGTAGACTTCTG-'3
*S. enterica*	*invA* gene	172	**Sen-1:** 5′-TTTCAATGGGAACTCTGC-'3 **Sen-2:** 5′-AACGACGACCCTTCTTTT-'3
*S. enterica*	*invA* gene	262	**Sal1598F:** 5′-AACGTGTTTCCGTGCGTAAT-'3 **Sal1859R:** 5′-TCCATCAAATTAGCGGAGGC-'3
*Listeria sp.*	*iap* gene	78	**iap31dF:** 5′-CAYCCGCWAGCACWGTAGTAGT-'3 **iap50dR:** 5′-GCGTCRACAGTWGTSCCHTT-'3
*Lactobacillus acidophilus*	16S-23S intergenic region	85	**LaF:** 5’-GAA AGA GCC CAA ACC AAG TGA TT-'3 **LaR:** 5’-CTT CCC AGA TAA TTC AAC TAT CGC TTA-'3
*Bifidobacterium bifidum*	16S-23S intergenic region	278	**BiBIF-1:** 5′-CCA CAT GAT CGC ATG TGA TTG-'3 **BiBIF-2:** 5′-CCG AAG GCT TGC TCC CAA A -'3

The qPCR was performed by using the ThermoFisher QuantStudio® 3 - 96-Well 0.2 ml Block. The qPCR cycle consists of the holding stage, the PCR stage, and the melt curve stage. In the holding stage, the initial denaturation step was at 50°C for 2 min and 95°C for 10 min. The PCR stage was for 40 cycles starting with denaturation. The denaturation step started at 95°C for 15 s, followed by annealing at 58°C for 15 s, and extension was at 75°C for 30 s. After the PCR stage, the melt curve stage started at 95°C for 15 s of denaturation, followed by 60°C for 1 min for annealing, and back to 95°C for 15 s for the final extension. Each set of primers went through at least three rounds of qPCR to check for accuracy and consistency. The relative abundance of selected bacteria for the feral and commercial chicken was obtained from normalized qPCR results at statistical significance (*P* < 0.01).

### Bioinformatics and Statistical Analysis

The raw sequencing reads were quality checked using FastQC (v0.11.9), and low-quality reads were filtered out from the subsequent analysis according to the following criteria: raw reads shorter than 110 nt and/or read with the length of the variable region shorter than 100 nt; reads lacking a perfect BLAST match to described barcodes; mismatches to at least one end of the 16S rRNA gene primers; and reads harboring more than 7% of low-quality bases (Phred score <20). Sequencing data were analyzed using the Quantitative Insights Into Microbial Ecology (QIIME v1.9.0). The paired-end reads were joined with a fast length adjustment of short reads (FLASH v1.2.11). After joining, the reads were then demultiplexed and quality filtered. Low-quality sequences from 16S rDNA sequencing data were trimmed based on the original raw data. Chimera checking was done using a module with the de novo method in USEARCH (v 6.1) software package (Caporaso et al., 2010). The operational taxonomic units (OTUs) were clustered at a threshold level of 97% sequence identity, and the sequences with the highest frequency were selected as the representative sequences of OTUs and annotated using the GreenGenes database (DeSantis et al., 2006). Shannon diversity and Simpson index were measures used for alpha-diversity as an indicator of evenness in community structure, richness, and the number of OTUs observed. The OTU abundance (Table S1) was normalized by the total number of reads in each sample and shown as relative abundance (Kumar et al. 2018). Bray-Curtis distance measure was used for the analysis of community similarity (beta**-**diversity). Phylogenetic Communities by Reconstruction of Unobserved States (PICRUSt, v1.1.3) was applied to predict the functional features of the intestinal microbiota based on the OTU table (Langille et al., 2013).

Statistical analyses were conducted using R software (v3.5.1). Differences in the abundance of genera, microbial alpha-diversity, and metabolic pathways were assessed by the Wilcoxon rank-sum test and considered significant at P < 0.05. Only those pathways and genera with more than 0.1% average abundance and only genera present in at least 20% of the samples were used for statistical analysis by Wilcoxon rank-sum test. Graph drawing and Principal coordinate analysis were plotted to visualize similarities or dissimilarities between the two types of birds using the ggplot2 package (Ito and Murphy, 2013). Principal coordinate analysis of a Bray-Curtis distance was performed in R v3.5.1 using the ade4 package (Zapala and Schork, 2006). Heatmaps were generated to show the presence of differentially abundant genera using the heatmap package. Linear Discriminant Analysis Effect Size (LEfSe Galaxy v1.0) algorithm with LDA effect size threshold of 2 (on a log_10_ scale) was applied to genus level data for potential biomarkers linked to both types of bird.

## RESULTS

### Next-Generation Sequencing

#### Sequencing Output Information.

A total of 1,038,227 sequences were obtained after quality filtering, where 549,230 and 488,997 sequences belong to feral and commercial chickens, respectively. The amplicon high-throughput sequencing results showed that there was, on average, 74,000 reads per sample, with 86% classified to the genus level.

#### Influence of Bird Type (Feral/Commercial) on Microbial Community Composition.

**Figure 1:**
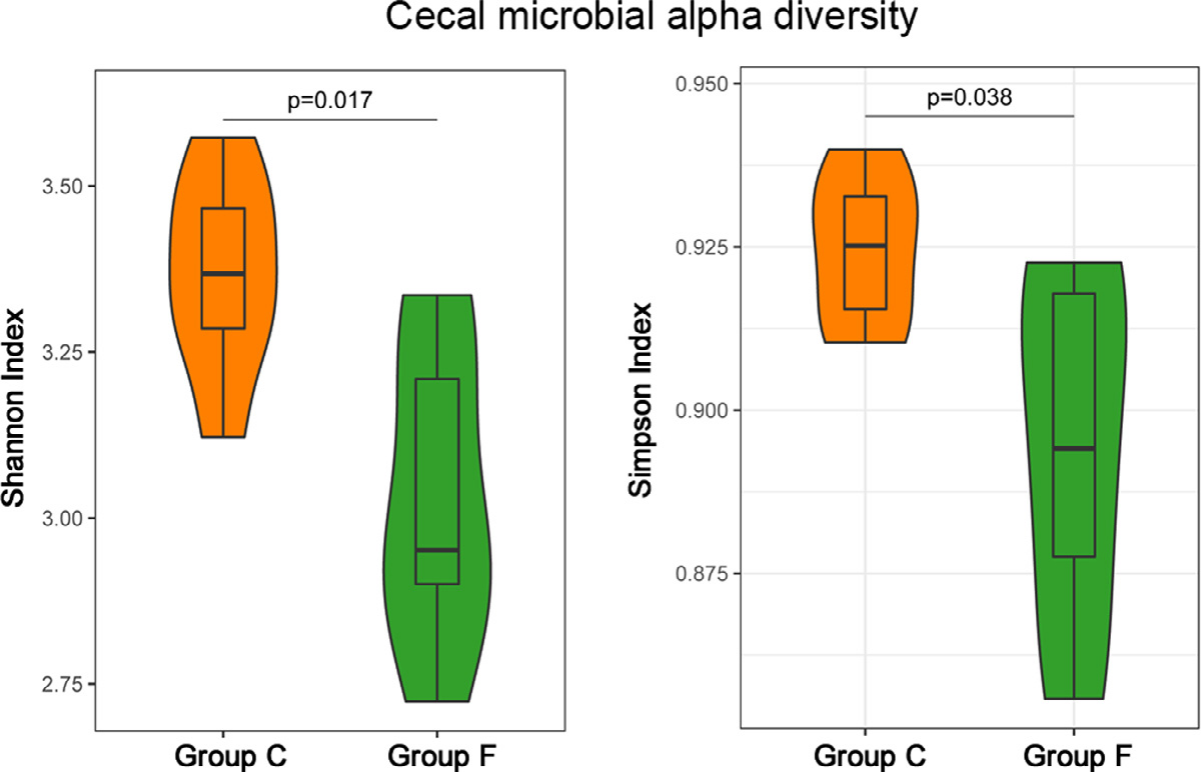
Alpha diversity: Shannon Index and Simpson Index for commercial and feral chicken samples. It illustrates the differences in species diversity within feral and commercial samples.

**Figure 2:**
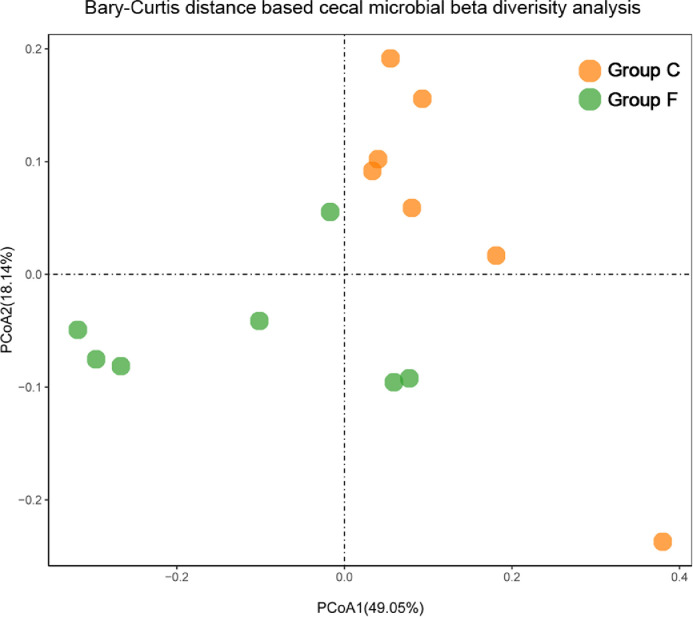
Beta diversity: Bray-Curtis distance based between community diversity analysis for commercial and feral chicken samples.

**Figure 3:**
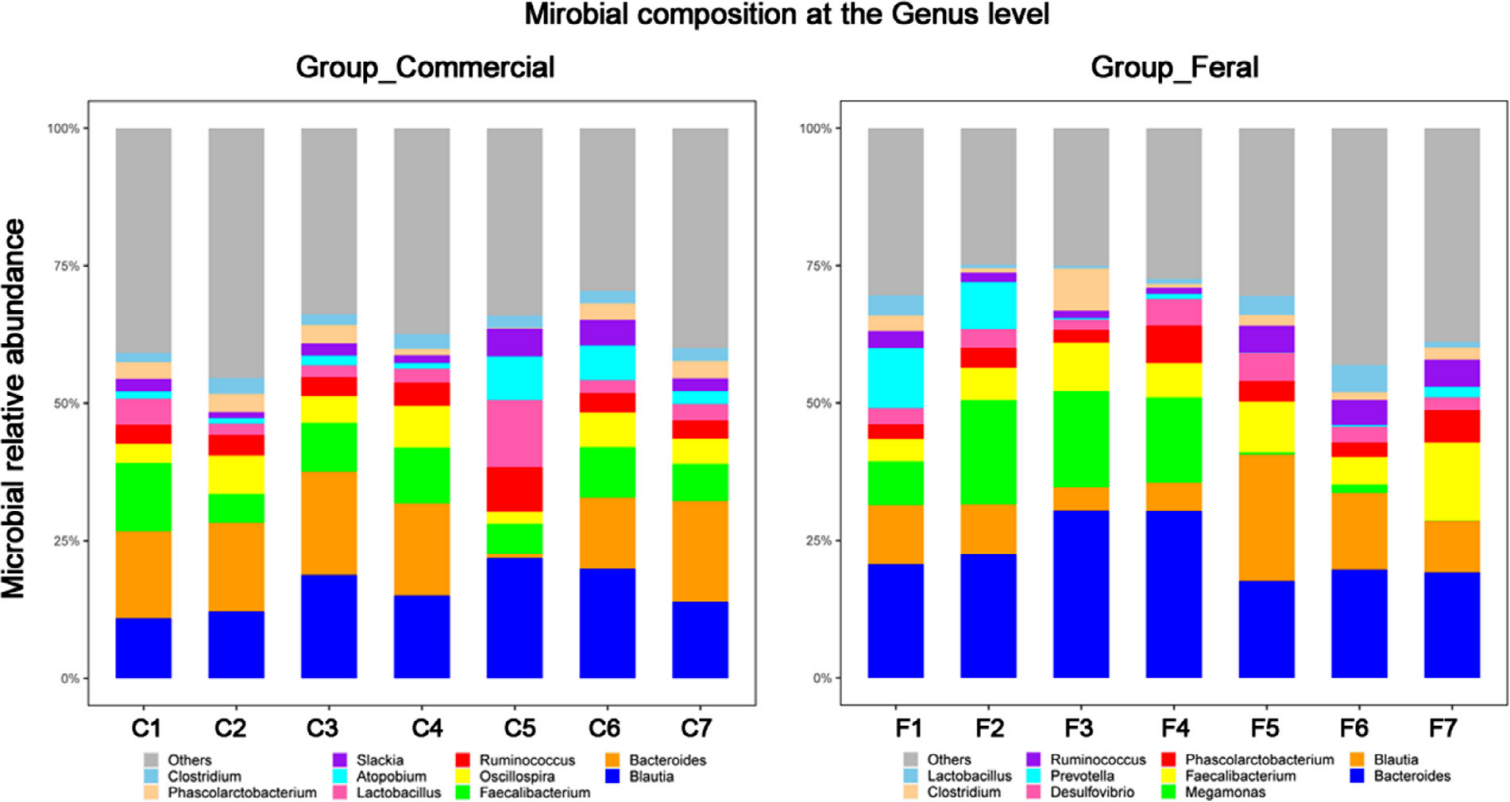
Microbial relative abundance at the genus level for both commercial and feral chicken samples identified from amplicon high-throughput sequencing results.

**Figure 4:**
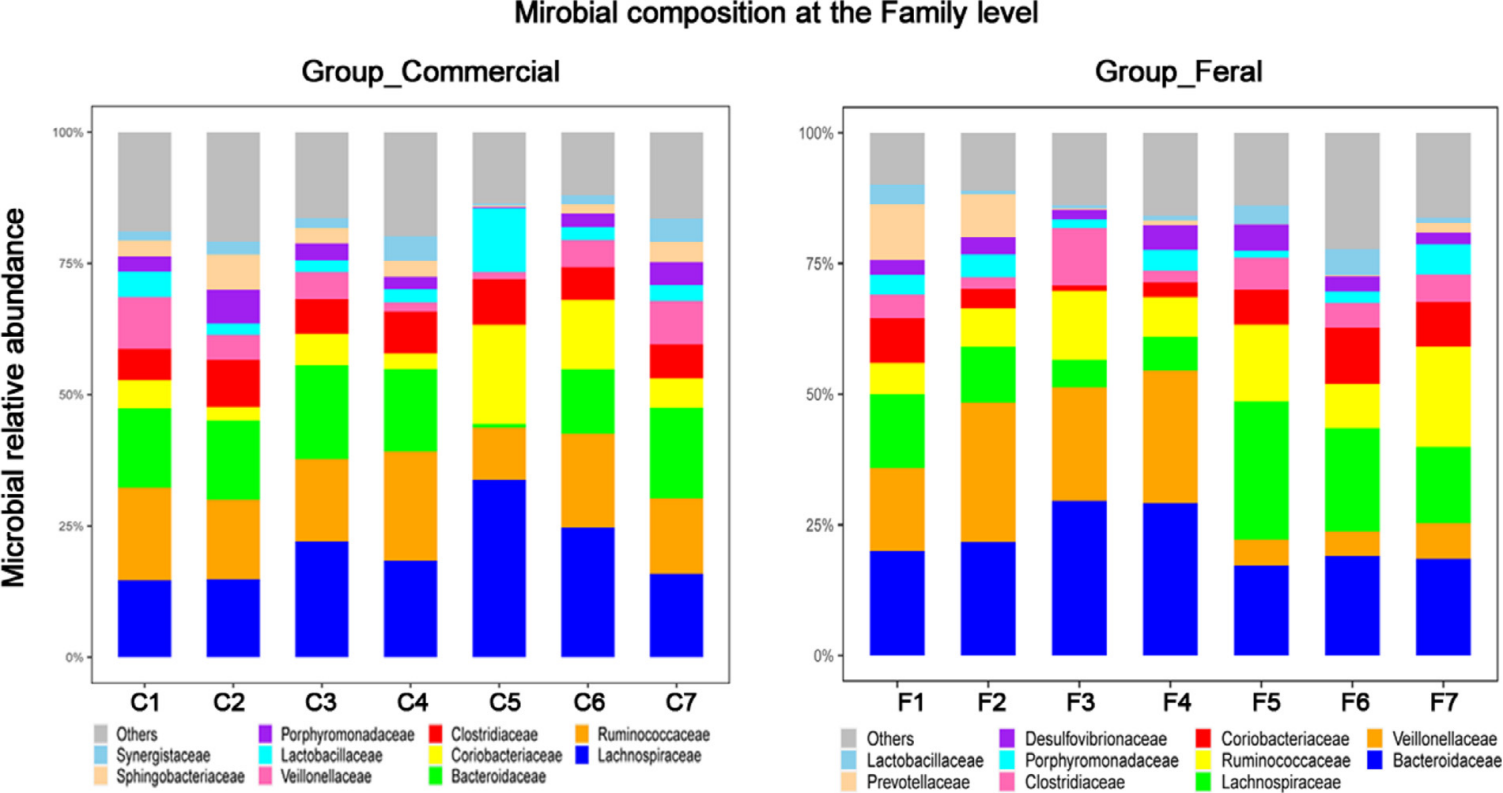
Microbial relative abundance at the family level for both commercial and feral chicken samples identified from amplicon high-throughput sequencing results.

**Figure 5:**
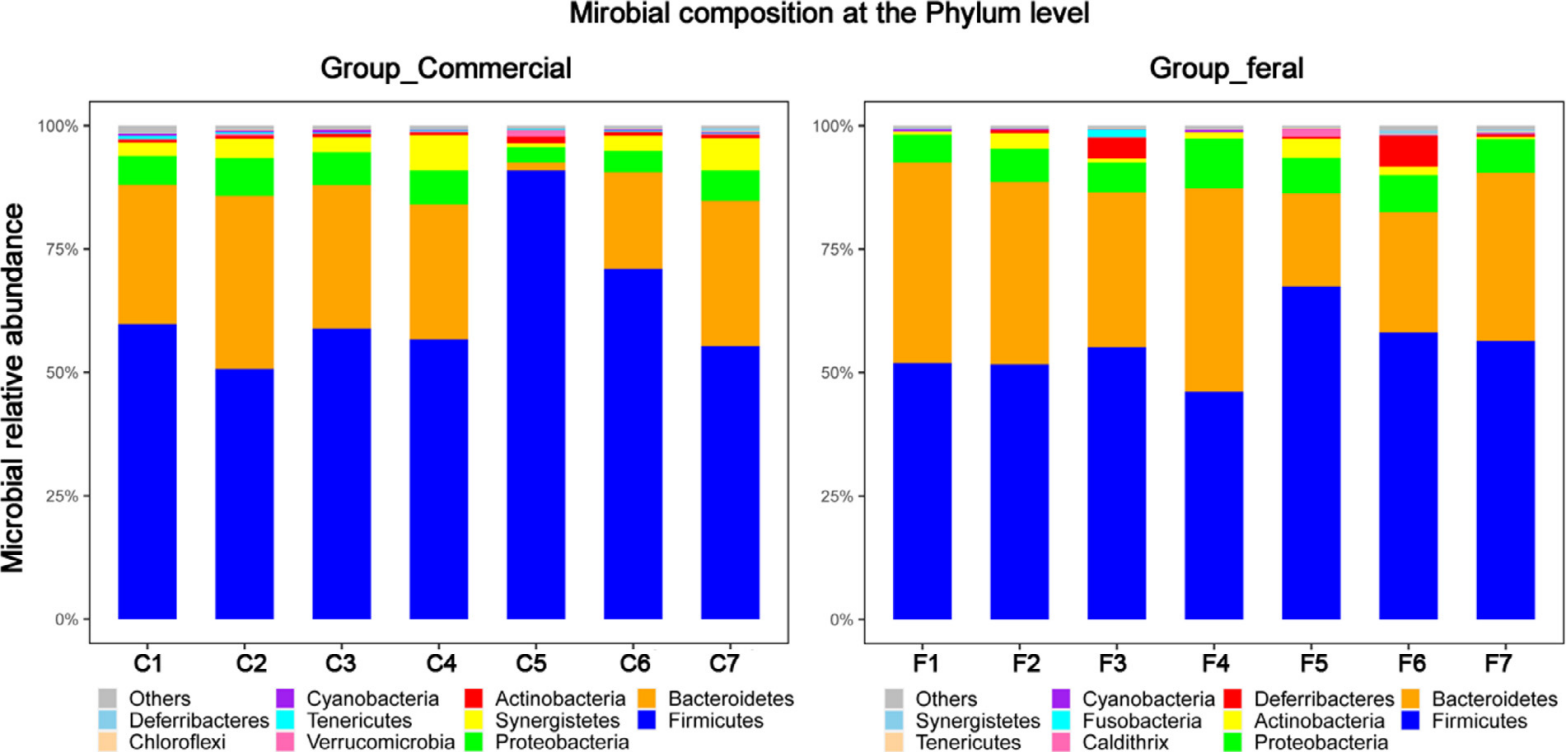
Relative abundance of bacteria in commercial chickens at the phylum level analyzed from the amplicon high-throughput sequencing results and GreenGenes database. It shows that the most abundant bacteria at the phylum level in both groups are Firmicutes, Bacteroidetes, and Proteobacteria.

**Figure 6:**
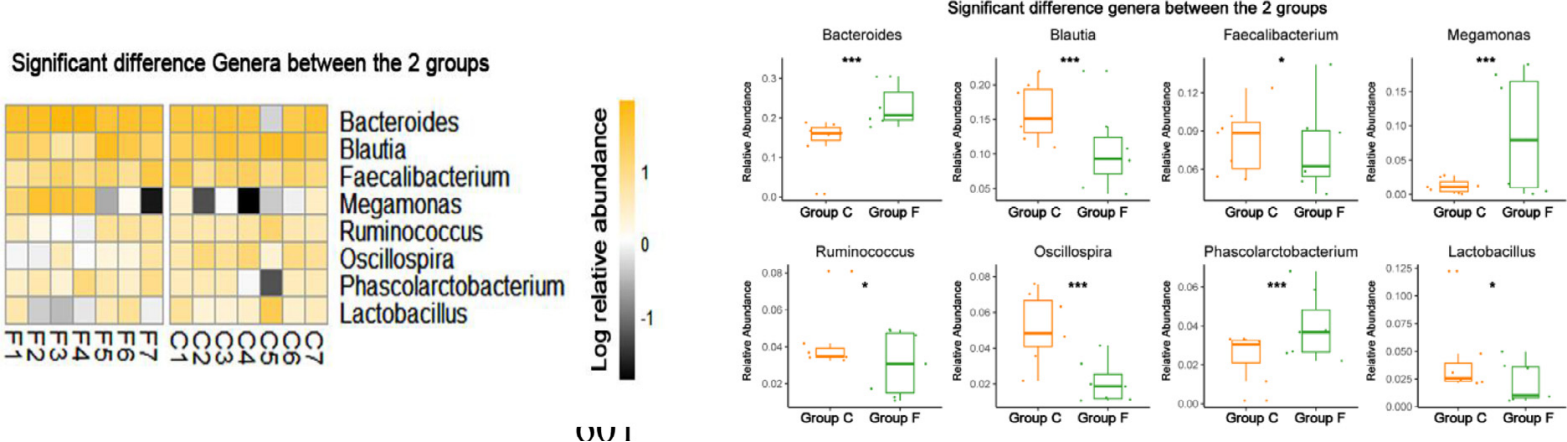
Left panel, the heatmap showing significant differences between bacteria in commercial and feral chicken groups at the genus level. Right panel, box plot quantified the significant difference genera between the commercial and feral chicken groups. “*” represent the significance at *P* < 0.05; “***” represent the significance at *P* < 0.001.

**Figure 7:**
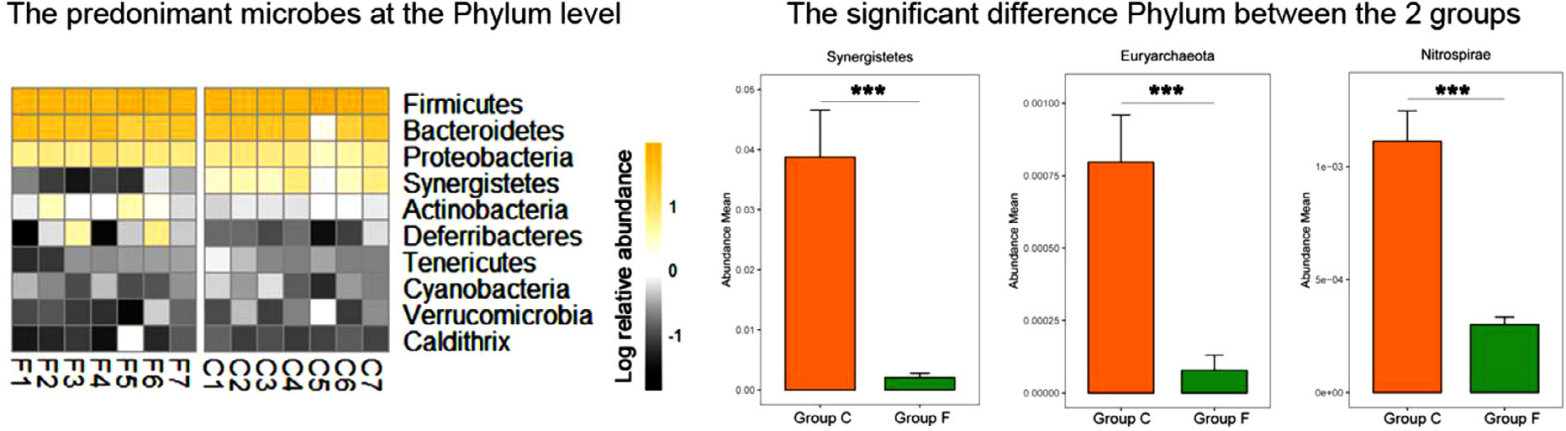
Left panel, the heatmap showing significant differences between bacteria in commercial and feral chicken groups at the phylum level. Right panel, box plot quantified the significant difference phylum between the commercial and feral chicken groups. “*” represent the significance at *P* < 0.05; “***” represent the significance at *P* < 0.001.

**Figure 8:**
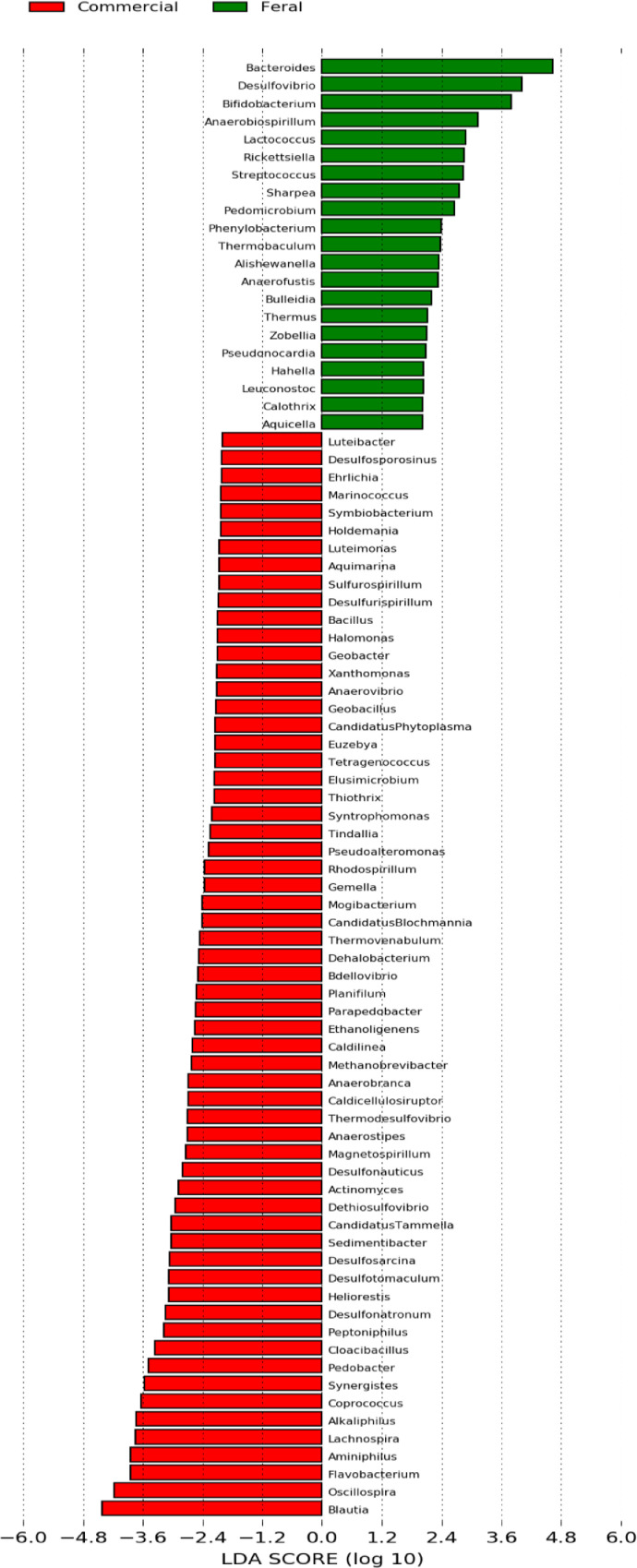
Histogram of the LDA scores computed for genera differentially abundant between commercial and feral chickens. Genera enriched in feral chickens are indicated with a positive LDA score, and genera enriched in commercial chickens have a negative score. The LDA score (>2 considered) indicates the effect size and ranking of each differentially abundant taxon.

The cecal bacterial community was inspected using 16S rRNA gene sequences. The alpha-diversity metrics of the cecal microbiota were significantly higher for commercial than feral chickens (Shannon diversity, *P* = 0.017; Simpson index, *P* = 0.038), as shown in Figure 1. The community of cecal bacteria colonizing feral and commercial chickens could be distinguished separately based on Bray-Curtis dissimilarity, as shown in Figure 2 (R^2^ = 0.29, *P* = 0.005; PERMANOVA). For the similarities between feral and commercial bacterial communities, the top 10 OTUs with the higher relative abundances are presented in Figures 3 to 5 at the genus, family, and phylum level, respectively. However, 8 of the OTUs were significantly different at the genus and phylum level between both groups of birds, as shown in Figure 6, Figure 7, respectively. Also, LEfSe analysis was performed to identify specific taxa that are different in abundance consistently by chicken type studied to shows potential biomarkers for both groups of birds at the genus level (Figure 8). For LEfSe, the Kruskal-Wallis test was done at alpha value 0.05, and LDA score >2 was used as a threshold.

#### Taxonomic Composition of Bacterial Community.

Phylum level relative abundance (Table S2) results showed that Firmicutes, Bacteroidetes, and Proteobacteria are the three most prevalent phyla for both feral and commercial chickens, making up at least 90% of bacteria present. In feral birds, the average percent abundance of Firmicutes was 55.3%, Bacteroidetes was 32.5%, and Proteobacteria was 7.1%. In commercial birds, the percent abundance of Firmicutes was 63.3%, Bacteroidetes was 24.4%, and Proteobacteria was 5.8%. The Firmicutes/Bacteroidetes ratio in the feral group was 1.7, compared to 2.6 in the commercial group. A closer microbial comparison at the phylum level revealed that the feral birds had a significantly higher log relative abundance of Caldithrix (0.003 vs. 0.001), Deferribacteres (0.017 vs. 0.0018), and Actinobacteria (0.017 vs. 0.007) than commercial birds. Similarly, commercial birds had a higher log relative abundance of Synergistetes (0.04 vs. 0.002), Verrucomicrobia (0.003 vs. 0.001), and Cyanobacteria (0.003 vs. 0.002) than feral birds (*P* < 0.05) (Figure 5).

To further identify the differences in microbial composition, the genus-level composition was explored (Table S3). The most abundant genera within the commercial chickens were *Bacteroides, Blautia*, and *Faecalibacterium*, whereas, for the feral chickens, it was *Bacteroides, Blautia*, and *Megamonas* (Figure 3). On average, feral chickens had a percent abundance of 23.0% *Bacteroides*, 10.8% *Blautia*, and 8.8% *Megamonas*. In comparison, commercial chickens had 14.2% *Bacteroides*, 16.1% *Blautia*, and 8.3% *Faecalibacterium* percent abundance. Also, the log relative abundance for *Ruminococcus* (0.04 vs. 0.03), *Oscillospira* (0.05 vs. 0.02), and *Lactobacillus* (0.04 vs. 0.02) was significantly higher for commercial birds, whereas *Phascolarctobacterium* (0.04 vs. 0.02) was higher for feral birds (*P* < 0.05) (Figure 8). The LEfSe analysis performed to identify biomarkers identified 82 OTUs at a threshold of LDA score >2 with significant differences between feral and commercial birds (*P* < 0.05). The cecal microbial sample showed 21 genus-level biomarker bacteria in feral birds, and 61 biomarker bacteria were present in commercial birds (with LDA scores >2) as potential biomarkers by LEfSe analysis for a distinction between feral and commercial birds.

#### Cecal Microbial Metabolic Pathway Prediction Analysis.

**Figure 9:**
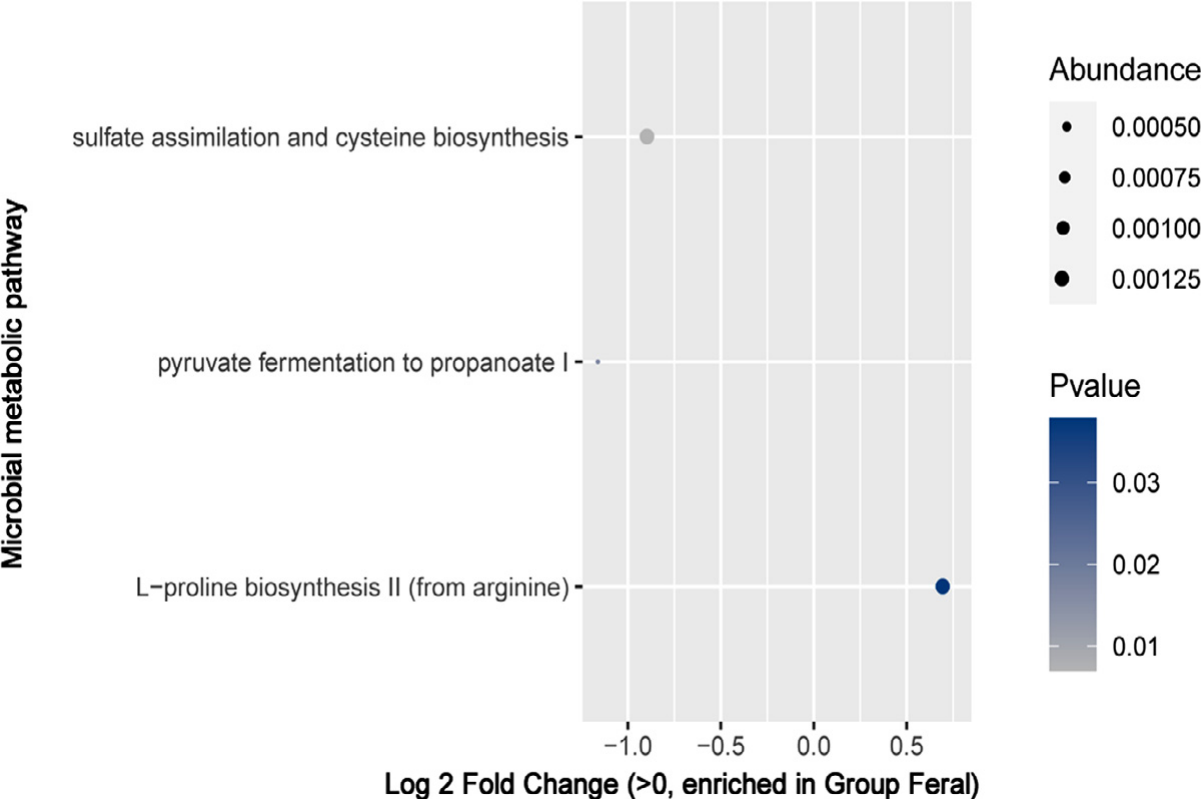
Significant enriched microbial metabolic pathways between the feral chicken and the commercial chicken groups.

For microbial metabolic pathway analysis, the PICRUSt software was used. The microbial metabolic pathway of sulfate assimilation, cysteine biosynthesis, and pyruvate fermentation to propanoate were enriched in the commercial birds (Figure 9). The L-proline biosynthesis pathway was significantly enriched in the feral chickens (Figure 9).

#### QPCR-Based Quantification of Target Bacteria.

**Figure 10:**
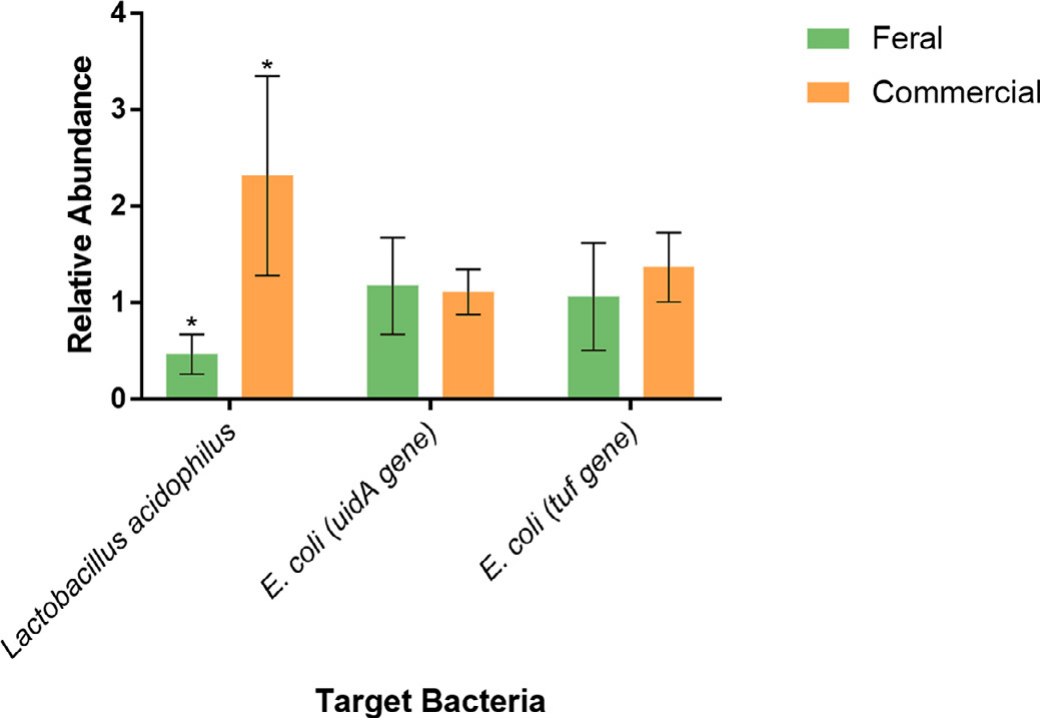
Relative abundance of selected bacteria for feral and commercial chickens by normalized qPCR results. The asterisk * signifies areas of statistical significance (*P* < 0.01).

The average CT values were taken for each primer pair to determine the abundance of the target gene amplified within the samples. Not all primer pairs could amplify the target gene, presumably because the specific gene was not present in the samples. As shown in Table 2, the target bacteria that were not able to be amplified were *Bifidobacterium bifidum, Salmonella enterica*, and *Listeria* spp*.* The target bacteria that were able to be amplified were *Lactobacillus acidophilus,* and *E. coli* with both the *uid*A and *tuf* gene. Table 2 shows that the feral chickens had a higher raw average CT value than the commercial chickens for all target genes amplified. When the average CT values were normalized to determine the relative abundance of each bacteria present, the qPCR data showed that the commercial chickens had a significantly higher (*P* < 0.01) relative abundance of *L. acidophilus* when compared to the feral chickens (Figure 10); on the other hand, there was no significant difference when comparing the relative abundance of *E. coli* between the 2 populations of birds.

**Table 2:** Average CT values for all primer pairs and samples. High CT values signify a lower abundance of bacteria present within a sample.

Target	Primer Pair	Feral	Commercial
Reference Gene (V3 16S rRNA)	p1/p2	14.148	12.291
*Lactobacillus acidophilus* (16S-23S intergenic region)	LaF/LaR	25.431	22.218
*E. coli* (*uid*A gene)	UAL754/UAR900	30.132	26.637
*E. coli* (*tuf* gene)	TEco553/TEco754	35.223	31.545
*Bifidobacterium bifidum (16S-23S intergenic region)*	BiBIF-1/BiBIF-2	UndetermiSned	Undetermined
*S. enterica* (*inv*A)	Sen-1/Sen-2	Undetermined	Undetermined
*S. enterica* (*inv*A)	Sal1598F/Sal1859R	Undetermined	Undetermined
*Listeria sp. (iap)*	iap31dF/iap50dR	Undetermined	Undetermined

Samples that did not amplify are denoted as “undetermined.”

## DISCUSSION

It has been well established that diet is a crucial factor that impacts the composition of gut microbiota. This study sought to characterize microbial community profiles from feral and commercial chickens, using the ceca as the location of the bacterial population. Methods such as amplicon high-throughput sequencing and qPCR were used to identify similarities and differences in the microbial profile between the feral and commercial chickens.

Different feed ingredients and eating behavior between feral and commercial chickens cause the differences in the gut microbiota diversity, composition, and overall community structure (Tan et al., 2019; Yadav and Jha, 2019). This study revealed an interesting finding that the Shannon and Simpson diversity index is less within the microbiome of feral chickens than in commercial chickens, which seems opposite to what one would expect from birds with a more diverse diet. One proposed reason for the lower alpha-diversity found in feral birds could be because the collected birds were already mature, and mature birds are found to have a relatively stable gut microbial diversity (Donaldson et al., 2017; Gong et al., 2019). Another potential reason for the decreased diversity could be due to an overabundance of a few dominant genera in matured feral birds. This prevalence of highly competitive microbial communities could also affect the richness and evenness that determines diversity (Rodrigues et al., 2020). Generally, high alpha-diversity is considered beneficial, but recent studies have shown that limited diversity is more desirable and advantageous as all microbes are not beneficial and reduce competition in less diverse birds (Reese and Dunn, 2018). The Bray-Curtis distance-based beta-diversity shows close clustering for commercial samples, whereas more inter-individual clustering was observed between samples of feral groups. This could be affected by the nature of the diet since birds in a commercial setup are provided with uniform commercial diets rich in readily digestible grains. In contrast, the feral bird's diet consists of fresh grass, insects, wild seeds, fruit, berries, and worms. This difference in beta-diversity could also be linked to a variation in the age of the feral birds (approximately 15–20 wk), which was much more diverse than the uniform age of the commercial birds (5 wk). Other contributing factors could be differences in behavioral patterns, the differences in the overall health, species differences, and the rearing environment (Clarke et al., 2014).

The amplicon high-throughput sequencing results allowed for a deeper examination of the most relatively abundant bacteria that are present in both groups. Similar to previous studies, the top three most abundant bacteria at the phylum level were Firmicutes, Bacteroidetes, and Proteobacteria (Stanley et al., 2013; Xu et al., 2016; Pandit et al., 2018), of which Firmicutes and Bacteroidetes consisted of more than 80% of all the microbiota. Both of these bacteria are linked to short-chain fatty acid (**SCFA**) production. Individually, Firmicutes aid in butyrate and propionate synthesis, whereas Bacteroidetes help in the synthesis of propionate, alpha-amylase, and other enzymes responsible for starch and polymeric substances breakdown (Polansky et al., 2016). Feral groups having higher Bacteroidetes may produce higher propionate that can be used as an energy source when they are unable to find adequate feed (Pandit et al., 2018). A high Firmicutes/Bacteroides ratio has been linked to an increase in body fat accumulation (Davis, 2016). Thus, the increased in the Firmicutes/ Bacteroidetes ratio (2.6 in commercial compared to 1.7 in feral) could be accounted for the heavier bodyweight of the commercial chickens (Davis, 2016). The higher ratio of Firmicutes/Bacteroides in commercial chickens could also be confounded with diet as commercial diet contains comparatively higher carbohydrates and a greater amount of grain-based balanced diet available to the commercial chickens, leading to a more rapid increase in weight. Furthermore, the scavenging nature of feral chickens gives them more access to fibrous feeds that could shape the gut microbiota with a greater prevalence toward *Bacteroides*, as seen in Figure 6. These bacteria are known as effective degraders of indigestible carbohydrates such as cellulose that could help maintain the growth of feral birds. This microbial shift also indicates that feral birds are still able to maintain bacterial phylogeny under different ecological circumstances. In support of these findings, the genus *Megamonas*, one that has also been shown to aid in the degradation of complex polysaccharides from plants, is present at a higher percentage in feral chickens. Additionally, *Megamonas* spp. are also known to uptake hydrogenases and promote SCFA production by acting as hydrogen sinks (Sergeant et al. 2014). These SCFA provide energy and inhibit acid-intolerant pathogens by reducing pH (Sergeant et al., 2014; Vaddu et al., 2021). These data show that there are more dominant bacteria within the feral chicken samples than the commercial chicken samples, and these dominant bacteria may serve essential roles in digestion and nutrient uptake. Studies by Tan et al. (2019) and Xu et al. (2016) also found similar predominant genera in feral animals. Furthermore, they have shown functional annotations for the digestive system and the biosynthesis of secondary metabolites. The PICRUSt results displayed a significantly higher enrichment of L-proline biosynthetic pathways in the feral chickens; on the other hand, there was a small increase in cysteine biosynthetic pathways in commercial birds. In other words, feral chickens appear to have a higher abundance of enzymes involved in amino acid and glycan metabolic pathways. These data suggest that the energy generated from protein metabolism in feral chickens is used for movement or activities rather than growth, thus increasing the meat quality of outdoor birds compared to commercial birds (Xu et al., 2016). Figure 3 shows that there is a similarity within the commercial chickens because it showed a very similar percentage for the three most abundant genera of bacteria – *Bacteroides, Blautia*, and *Faecalibacterium*. Additionally, LEfSe analysis found *Blautia*, a biomarker for propionate and butyrate production, in the commercial chicken group. There was also a noticeable variation between the feral chickens, as there were differences in the percentages of the three most abundant genera of bacteria (Figure 3). Also, based on LEfSe analysis, the bacterial genus *Oscillospira* was more abundant in commercial birds. It has been shown that *Oscillospira* plays a role in butyrate production, as well as the utilization of glucuronate, a common animal-derived sugar, and its presence is reduced in cases of inflammation (Nielsen et al., 2014). Similarly, the commercial samples have a significantly greater percentage of the target bacteria *Lactobacillus* (Figure 6). A significant biomarker found in the feral chickens was *Desulfovibrio*, which has been shown to aid in scavenging free hydrogen released by many anaerobes during fermentation. A similar function can be performed by *Blautia* in commercial birds.

The data obtained by qPCR correlates with the amplicon high-throughput sequencing results in several ways. As shown in Table 2, primers specific to *Salmonella enterica*, and *Listeria* did not produce an amplicon, suggesting that they were not present, at least in quantities high enough to detect. The bacterium *Bifidobacterium* was present in the feral group according to the sequencing data, and these probiotic bacteria colonize the mucosal surface and provide numerous nutritional and health benefits (Matsuki et al., 2003). Overall, the feral chicken samples displayed a numerically higher raw CT value than the commercial samples (Table 2), which indicates the target bacteria that were amplified are less abundant in the feral chickens than in the commercial chickens. When normalized, the relative abundance of *E. coli* (*tuf & uidA* gene) between feral and commercial chickens was not significantly different (Figure 10). This corresponds with the amplicon high-throughput sequencing results, which show that *E. coli* is present in negligible amounts in both feral and commercial chickens. On the other hand, *Lactobacillus acidophilus* was statistically less abundant in feral chickens than in commercial chickens (Figure 10). While this data is species-specific, it also corresponds to the previously obtained amplicon high-throughput sequencing results that show that the genus *Lactobacillus* is slightly less abundant in feral chickens than in commercial chickens.

In conclusion, there was a greater percentage of target bacteria in the Hawaiian feral chickens than the commercial chickens, albeit with lower diversity but with different pathways enriched. The microbial diversity seen within the feral chickens could be due to their irregular diet and inconsistent living conditions. In contrast, commercial chickens are exposed to a more consistent diet and living conditions, explaining the relative similarity of the microbial communities found in their ceca. Creating a microbial community profile for feral and commercial chickens and identifying the biomarkers for their differences could aid in developing breeds that are more efficient in growth performance as well as more resistant to pathogens. From this study, we can also conclude that the microbiota in feral chickens are more directed to complex feed digestion and obtaining energy. In contrast, commercial chickens’ microbes have divided functions between maintaining gut health and enhancing growth to maximize their body weight in a short time to their genetic potential. Future studies can focus on decreasing the variables and confounding factors to recognize the causes of the differences in the bacteria present and their relative abundance with more specificity. These include modes of action, an interaction between different microbes in the community and with its host, linkage to the host body system, and how exactly the microbiome plays a role in changing the host's phenotypic traits or physiological status.

## Appendix Supplementary materials

Image, application 1

Image, application 2

Image, application 3
